# The Innate Immune Response to Herpes Simplex Virus 1 Infection Is Dampened in the Newborn Brain and Can Be Modulated by Exogenous Interferon Beta To Improve Survival

**DOI:** 10.1128/mBio.00921-20

**Published:** 2020-05-26

**Authors:** Daniel Giraldo, Douglas R. Wilcox, Richard Longnecker

**Affiliations:** a Department of Microbiology and Immunology, Northwestern University Feinberg School of Medicine, Chicago, Illinois, USA; b Department of Neurology, Brigham and Women’s Hospital, Boston, Massachusetts, USA; c Department of Neurology, Massachusetts General Hospital, Boston, Massachusetts, USA; d Department of Neurology, Harvard Medical School, Boston, Massachusetts, USA; Princeton University

**Keywords:** herpes simplex virus, interferon, newborn, viral encephalitis

## Abstract

Newborns are particularly susceptible to severe forms of herpes simplex virus 1 (HSV-1) infection, including encephalitis and multisystemic disseminated disease. The underlying age-dependent differences in the immune response that explain this increased susceptibility relative to the adult population remain largely understudied. Using a murine model of HSV-1 infection, we found that newborn mice are largely susceptible to intracranial and intraperitoneal challenge while adult mice are highly resistant. This age-dependent difference correlated with differential basal-level expression of components of innate immune signaling pathways, which resulted in dampened interferon (IFN) signaling in the newborn brain. To explore the possibility of modulating the IFN response in the newborn brain to recapitulate the adult phenotype, we administered exogenous IFN-β in the context of disseminated HSV-1 infection. IFN-β treatment resulted in significantly increased survival and delayed viral neuroinvasion in the newborn. These effects were associated with changes in the type I IFN response in the brain, reduced viral replication in the periphery, and the stabilization of the blood-brain barrier (BBB). Our study reveals important age-dependent differences in the innate immune response to HSV-1 infection and suggests a contribution of the BBB and the brain parenchyma in mediating the increased susceptibility to HSV-1 infection observed in the newborn. These results could provide the basis for potential new therapeutic strategies for life-threatening HSV-1 infection in newborns.

## Abstract (precis)

Herpes simplex virus (HSV) is a ubiquitous human pathogen affecting 50 to 80% of the population in North America and Europe. HSV infection is commonly asymptomatic in the adult population but can result in fatal encephalitis in the newborn. Current treatment with acyclovir has improved mortality in the newborn; however, severe neurologic sequelae are still a major concern following HSV encephalitis. For this reason, there is a critical need to better understand the underlying differences in the immune response between the two age groups that could be used to develop more effective treatments. In this study, we investigated differences in the innate immune response to viral infection in the brains of newborn and adult mice. We found that, similar to humans, newborn mice are more susceptible to HSV infection than the adult. Increased susceptibility was associated with dampened innate immune responses in the newborn brain that could be rescued by administering interferon beta.

## INTRODUCTION

Herpes simplex virus (HSV) is a ubiquitous human pathogen affecting 50 to 80% of the population in North America and Europe (1, 2). HSV establishes a lifelong infection by remaining latent in the trigeminal ganglia and periodically reactivating (3, 4). Disease outcomes of HSV infection can range from asymptomatic viral shedding to life-threatening conditions, including herpes simplex encephalitis (HSE) and multiorgan infection through hematogenous spread affecting multiple organs, resulting in disseminated disease (5). Despite the high seroprevalence of HSV in the adult population, these more severe forms of disease are rare. Conversely, newborns are particularly susceptible to severe disease, with 50% of those infected developing HSE or disseminated disease (6). HSV-1 is the most common etiological agent of sporadic viral encephalitis (7) and has a higher risk of transmission in the newborn than HSV-2 (5). The striking difference in disease outcomes between newborns and adults suggests an age-dependent susceptibility to infection associated with the host immune response.

Several *in vitro* and *in vivo* studies have pointed to the type I interferon (IFN) response as a critical pathway involved in the early immune response to infection (8–10). Multiple pattern recognition receptors, such as toll-like receptor 3 (TLR-3) (11) and TLR-9 (12), as well as their downstream effectors, have been shown to be involved in controlling viral replication in the central nervous system in murine models of infection (13). Importantly, human genetic studies have linked multiple mutations in the type I IFN pathway to the high incidence of HSE in children and adults (14–16). However, the high rates of severe disease observed in the newborn likely cannot be entirely attributed to inborn errors.

Here, we show that interferon α/β receptor (IFNAR) signaling provides protection against infection in the adult but is insufficient to protect the newborn. Increased susceptibility in the newborn was associated with differential basal levels of type I IFN response components in the brain. These differences did not reflect a global downregulation of this pathway in the brain. While certain components, such as IFNAR, were found to be downregulated in the newborn, other components of this pathway showed no difference or were actually higher in the newborn. Most interestingly, we found that treatment with exogenous interferon beta (IFN-β) completely protected the newborn from infection. Increased survival was associated with the upregulation of cGAS in the brain parenchyma, delayed spread to the central nervous system (CNS), and stabilization of the blood-brain barrier during disseminated disease.

## RESULTS

### IFNAR signaling protects the adult brain from HSV infection but is insufficient for protection in the newborn.

**FIG 1:**
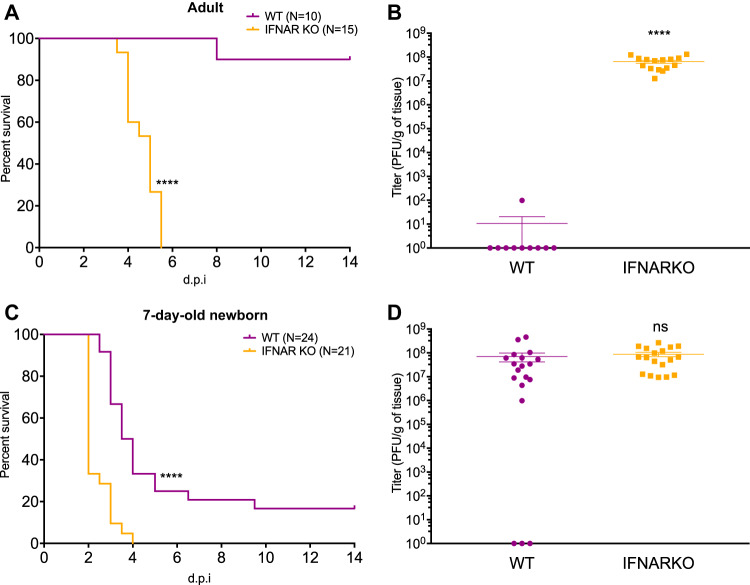
IFNAR protects against mortality and decreases viral replication during HSV-1 CNS infection in the adult but only prolongs survival in the newborn. (A and B) Survival (A) and viral titer of brains at mortality or experimental endpoint at day 14 (B) of adult WT or IFNARKO mice inoculated i.c. with 10^4^ PFU of HSV-1 KOS. d.p.i, days postinfection. (C and D) Survival (C) and viral titer of brains at mortality or day 14 (D) of 7-day-old (P7) WT or IFNARKO mice inoculated i.c. with 10^4^ PFU of HSV-1 KOS. (*, *P* ≤ 0.05; **, *P* ≤ 0.01; ***, *P* ≤ 0.001; ****, *P* ≤ 0.0001; ns, not significant. All error bars represent SEM.)

To determine the role of IFNAR signaling during HSV-1 infection of the CNS in the newborn and the adult, we inoculated wild-type (WT) and IFNAR knockout (IFNARKO) newborn and adult mice with 10^4^ PFU of WT HSV-1 strain KOS intracranially (i.c.). WT adult mice were highly resistant to infection, with only one mouse succumbing to infection, whereas all IFNARKO adults died, on average, 5 days postinfection (Fig. 1A). IFNARKO adult mice had high viral loads at mortality, while WT adult mice had undetectable viral loads in the brain at day 14. The single WT adult mouse that died prior to the experimental endpoint at day 14 had viral titers 6-fold lower than those of IFNARKO mice (Fig. 1B). Conversely, the survival of WT mice decreased to 17% in the WT newborn, while IFNARKO newborn mice displayed 100% mortality (Fig. 1C). Interestingly, there was a significant difference in survival between WT and IFNARKO newborn mice, with a median difference of 1.75 days, suggesting that IFNAR signaling prolongs survival in the newborn but is insufficient to provide protection from mortality. Viral titers were not significantly different at mortality between WT and IFNARKO newborn mice (Fig. 1D).

### The type I IFN response is differentially regulated throughout development.

**FIG 2:**
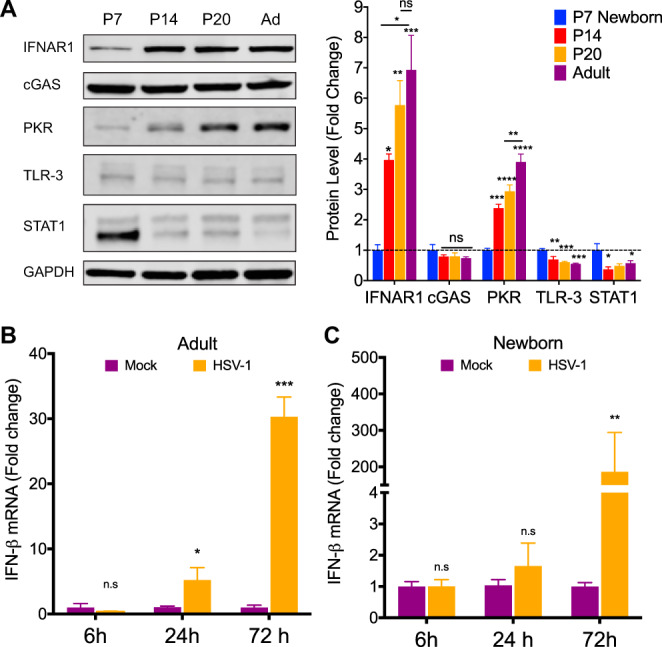
Type I IFN response components are differentially expressed in the brain throughout development. (A) Representative immunoblots (left) and densitometry (right) of whole-brain homogenates from uninfected 7-day-old (P7), 14-day-old (P14), 20-day-old (P20), and adult (Ad; 8- to 10-week-old) mice. Protein levels of IFNAR1 and PKR were significantly lower in 7-day-old mice and gradually increased during the first weeks after birth to adult levels. TLR-3 and STAT1 (STAT1α [91 kDa] and STAT1β [84 kDa]) protein levels were higher in 7-day-old mice than in other age groups. (B and C) IFN-β mRNA levels quantified by qPCR following i.c. inoculation with 10^4^ PFU HSV-1 (KOS) of P7 newborn (B) and adult (C) mice at different time points. Samples were normalized to GAPDH gene expression for each age group. (*N* = 3 to 6 for each experiment. *, *P* ≤ 0.05; **, *P* ≤ 0.01; ***, *P* ≤ 0.001; ****, *P* ≤ 0.0001. All error bars represent SEM.)

We next investigated the possibility that increased susceptibility to HSV-1 in the newborn was the result of differences in the basal levels of cellular proteins in the type I IFN response pathway in the newborn brain compared to that of the adult. Notably, IFNAR levels were 7-fold lower in the 7-day-old brain than in the adult (Fig. 2A), consistent with prior reports (17). Interestingly, IFNAR steadily increased during the first weeks of life and was already 4-fold higher in the 14-day-old-brain and not significantly different from the adult at weaning age (20 days old [P20]) (Fig. 2A). We observed a similar pattern of expression for protein kinase R (PKR), a major IFN-stimulated gene (ISG) known to play a crucial role in controlling HSV replication, with a 4-fold increase between the newborn and the adult brain (Fig. 2A). Importantly, our results did not suggest a global downregulation of the type I IFN response components in the newborn brain. The protein level of TLR-3, which recognizes double-stranded RNA and is upstream of IFNAR, was 2-fold higher in the newborn brain than in the adult (Fig. 2A). This finding is in agreement with previous reports showing the important role of TLR-3 during neurodevelopment (18). STAT1, the transcription factor directly downstream of IFNAR, was present in P7 newborns at levels 3-fold higher than that in the adult brain (Fig. 2A). STAT1 is expressed as two active splicing variants, STAT1α and STAT1β, involved in type I IFN signaling (19). Finally, cGAS, a major nucleic DNA sensor, did not display differences in levels across the four age groups (Fig. 2A).

### The type I IFN response to HSV-1 infection is diminished in the newborn compared to that in the adult.

We next explored whether the differential regulation of the basal levels of the type I IFN response components in the newborn brain correlated with an impaired response to HSV-1 infection. We monitored IFN-β mRNA levels in the brains of mock- and HSV-1-infected newborn and adult mice following intracranial challenge by quantitative reverse transcription-PCR (qRT-PCR). IFN-β mRNA levels were unchanged 6 h postinfection (hpi) but were 6-fold higher in the adult brain 24 hpi and continued to increase 30-fold by 72 hpi (Fig. 2B). Interestingly, newborn mice failed to upregulate IFN-β mRNA expression following intracranial challenge early during infection but displayed surprisingly high levels of IFN-β mRNA with an average 186-fold increase in the brain 72 hpi (Fig. 2C). This time point corresponds with the onset of mortality in the newborn, where viral titers in the brain can be as high as 10^8^ PFU/g of tissue (Fig. 1D), which suggests the newborn brain is capable of inducing high levels of type I IFN in response to a strong stimulus.

**FIG 3:**
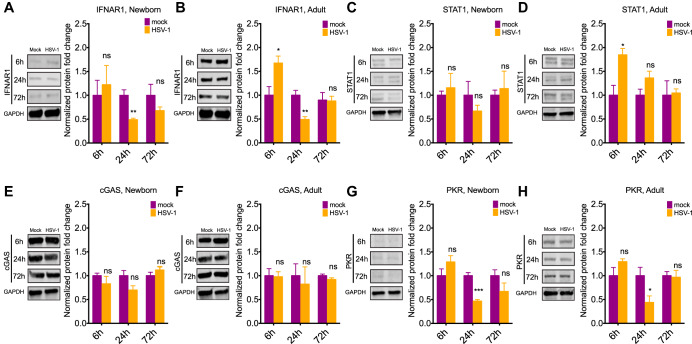
Type I IFN response components are rapidly upregulated in the adult but not in the newborn brain following HSV-1 infection. (A to H) Representative immunoblots (left) and densitometry (right) of IFNAR1 (A and B), STAT1 (C and D), cGAS (E and F), and PKR (G and H) in the brains of newborn and adult mice following i.c. inoculation with 10^4^ PFU HSV-1 analyzed by Western blotting at different time points. Samples were normalized to GAPDH levels for each age group at each time point. Significance denotes comparison of normalized values to the corresponding mock infection for each time point and age group. (*N* = 3 to 4 for each experimental condition. *, *P* ≤ 0.05; **, *P* ≤ 0.01; ***, *P* ≤ 0.001; ****, *P* ≤ 0.0001. All error bars represent SEM.)

A major feature of the type I IFN response is the upregulation of its different components in response to viral infection. We investigated whether increased susceptibility to HSV-1 infection in the newborn was also associated with differential regulation of the type I IFN pathway in response to infection. We collected brain tissue from newborn and adult mice at different time points following intracranial infection and determined protein levels of relevant components of this pathway by Western blotting. We found that IFNAR1 levels doubled in the adult brain after only six 6 h postinfection but did not significantly change in the newborn brain (Fig. 3A and B). IFNAR1 levels decreased 24 h postinfection in the adult and the newborn, consistent with a previously described negative feedback loop that prevents sustained IFNAR signaling (20, 21) (Fig. 3A and B). STAT1 levels also increased 2-fold in the adult brain 6 h postinfection but remained the same in the newborn brain (Fig. 3C and D). Interestingly, no significant changes were detected in PKR or cGAS expression throughout infection in the newborn and adult brain besides a decrease in PKR levels at 24 h (Fig. 3E to H).

### Exogenous IFN-β treatment completely protects the newborn from disseminated disease.

Our findings suggested that increased susceptibility in the newborn was associated with decreased basal levels of IFNAR1 and PKR in the brain, a failure to induce IFN-β production, and the inability to mount an effective type I IFN response in the brain. From the observation that IFNAR signaling prolonged survival in the newborn but failed to provide protection, we hypothesized that the administration of exogenous IFN-β could be used to modulate the innate immune response in the newborn and provide protection against HSV-1 infection.

**FIG 4:**
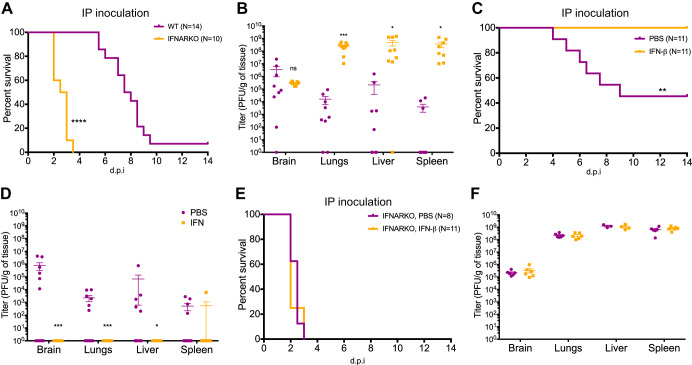
Recombinant murine IFN-β treatment protects the newborn from disseminated HSV-1 infection in an IFNAR-dependent manner. (A and B) Survival (A) and titer at mortality (B) of newborn WT or IFNARKO mice inoculated i.p. with 10^5^ PFU of HSV-1 KOS. IFNAR expression prolonged survival of newborn mice but failed to protect against mortality or control viral replication in the CNS. (C and D) Survival (C) and titer at mortality (D) of newborn WT mice inoculated i.p. with 10^5^ PFU of HSV-1 KOS and given daily doses of 10^4^ IU of recombinant murine IFN-β or vehicle control (PBS). (E and F) Survival (E) and titer at mortality (F) of newborn IFNARKO mice inoculated i.p. with 10^5^ PFU of HSV-1 KOS and given daily doses of 10^4^ IU of recombinant murine IFN-β or vehicle control (PBS). (*, *P* ≤ 0.05; **, *P* ≤ 0.01; ***, *P* ≤ 0.001; ****, *P* ≤ 0.0001. All error bars represent SEM.)

To test this hypothesis, we switched to a more physiologically relevant model of infection that better models HSV-1 dissemination to the brain in a natural infection. Although HSV-1 infection in the adult brain is thought to occur through transneural spread from peripheral ganglia, data from neonatal HSV-1 infections suggest that hematogenous spread during primary infection is the main route of infection in the newborn (22, 23). We used intraperitoneal (i.p.) inoculation to replicate hematogenous spread in the newborn (22). WT and IFNARKO newborn mice were infected i.p., and, similar to IC inoculation, IFNAR1 signaling prolonged survival but failed to protect the newborn. Newborn mice lacking IFNAR1 had a 100% mortality rate with a median survival of 2.75 days, while newborn WT mice had an 83% mortality rate with a median survival of 7.75 days (Fig. 4A). Interestingly, although viral titers in peripheral organs were around 5 orders of magnitude higher in IFNARKO mice, there was no difference in viral titers in the brain between WT and IFNARKO mice at mortality (Fig. 4B).

Type I IFNs (IFN-β and IFN-α) are the main cytokines induced by the type I IFN response and the cognate ligands of IFNAR (24). IFN-β is one of the most bioactive members of this family and is encoded by a single gene, compared to 14 and 13 for all the IFN-α variants in mice and humans, respectively (25, 26). IFN-β previously was used to treat several different diseases, including multiple sclerosis (MS) (27, 28), chronic hepatitis C infection (29–31), and some forms of cancer (32). We hypothesized that since insufficient IFNAR signaling correlated with decreased survival in the newborn, administering exogenous IFN-β provides protection against HSV-1 infection in the newborn. To test this, we administered daily doses of 10,000 IU IFN-β or vehicle i.p. to newborn WT mice starting the day before infection. We then challenged these mice with 10,000 PFU HSV-1 (KOS) i.p. IFN-β treatment provided complete protection from HSV-1 infection in the newborn (Fig. 4C). Phosphate-buffered saline (PBS)-treated WT newborns displayed increased survival compared to that of untreated WT newborns (Fig. 4A), which in part could be due to decreased dehydration from the administration of fluids (PBS) during infection. With the exception of one spleen, viral loads were undetectable in all peripheral organs and the brain 14 days postinfection in newborn mice treated with IFN-β (Fig. 4D). To ensure IFN-β was acting through the canonical type I IFN pathway, we repeated the same experiment using newborn IFNARKO mice. IFN-β treatment did not provide any survival advantage to IFNARKO compared to vehicle-treated mice (Fig. 4E) and did not affect replication in the periphery or the brain at the time of mortality (Fig. 4F), confirming an IFNAR1-dependent role for increased survival in mice treated with IFN-β.

### IFN-β treatment alters the type I IFN response pathway in the brain parenchyma and reduces peripheral viral replication.

**FIG 5:**
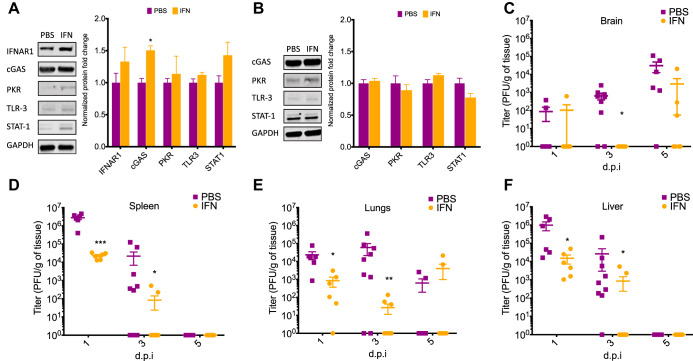
IFN-β treatment increases levels of cGAS in the newborn brain in an IFNAR-dependent manner and delays neuroinvasion during disseminated HSV-1 infection. Representative immunoblots (left) and densitometry (right) of whole-brain homogenates from uninfected WT (A) or IFNARKO (B) newborn mice treated for 3 days with either 10^4^ IU of mouse IFN or PBS (vehicle-only) control (*N* = 4 to 8 in each group). Newborn WT mice were inoculated i.p. with 10^5^ PFU of HSV-1 (KOS) and given daily doses of 10^4^ IU of recombinant murine IFN or vehicle control (PBS). (C to F) Liver (C), lungs (D), spleen (E), and brain (F) were collected on days 1, 3, and 5 postinfection, and viral loads were determined by standard plaque assay. (*, *P* ≤ 0.05; **, *P* ≤ 0.01; ***, *P* ≤ 0.001; ****, *P* ≤ 0.0001. All error bars represent SEM.)

We wanted to determine the extent to which exogenous IFN treatment controls peripheral viral replication and induces changes in the type I IFN pathway in the brain. To test this, we treated naive newborn WT mice with IFN-β or vehicle for 3 days and then collected brain tissue to analyze changes in type I IFN component levels. We found that peripheral IFN-β treatment induced a significant upregulation of cGAS in the newborn brain (Fig. 5A). We observed a trending increase in the levels of IFNAR1 and STAT1 following IFN-β treatment, similar to our findings in the adult brain following infection (Fig. 3B and D). These results suggest that IFN-β treatment has an effect on the brain parenchyma and is modulating the type I IFN response in the newborn brain. We repeated the same experiment using IFNARKO mice and found no difference in any of the type I IFN components analyzed, indicating these changes are IFNAR1 dependent (Fig. 5B).

Our results suggest that IFN-β treatment provides protection in the newborn by modulating the type I IFN response in the brain. To determine whether IFN-β treatment blocked viral replication in the brain or was preventing the spread of HSV-1 to the CNS by reducing viral replication in the periphery, we tracked viral replication in the periphery and the brain following i.p. inoculation of newborn WT mice. IFN-β treatment significantly reduced viral loads in the liver, lungs, and spleen at days one and three postinfection (Fig. 5C and D and E). Interestingly, viral loads in the periphery decreased over time in both vehicle- and IFN-β-treated mice but increased over time in the brain of vehicle-treated mice (Fig. 5F). Even though we were able to detect HSV-1 in the CNS of vehicle-treated mice as early as day one postinfection, we could not consistently detect HSV-1 in the CNS of IFN-β-treated mice until day five postinfection, with the exception of one mouse with detectable levels 1 day postinfection (Fig. 5F). Although viral replication was decreased in the periphery following IFN-β treatment compared to that of mock treatment, viral loads overall remained high in peripheral organs, particularly during the early stages of infection. However, we observed significantly decreased CNS invasion of the virus relative to that of vehicle-treated mice. Importantly, active viral replication in the brains of IFN-β-treated mice later in infection suggests that changes previously observed in the IFN pathway (Fig. 5A) in response to IFN treatment play a role in controlling viral replication in the brain and improving overall survival.

### IFN-β treatment stabilizes the blood-brain barrier during HSV-1 disseminated disease in the newborn.

**FIG 6:**
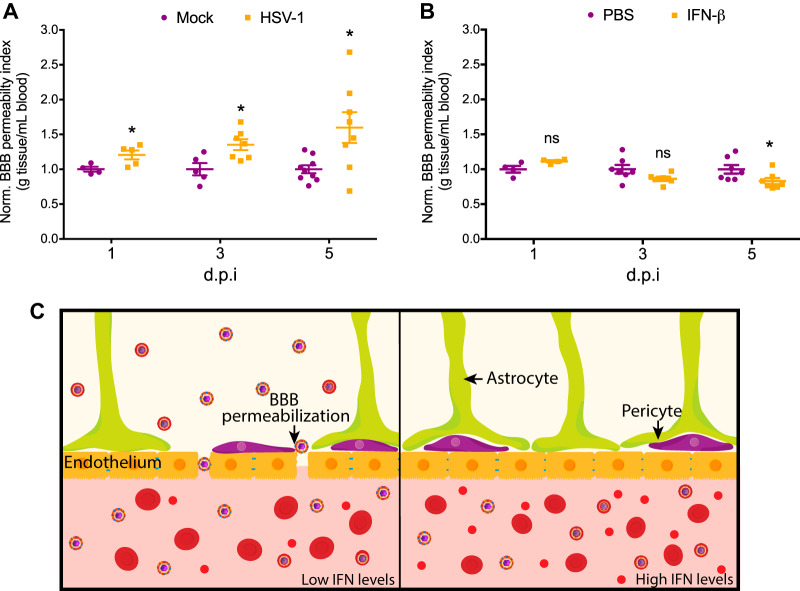
HSV-1-induced breakdown of the blood-brain barrier during disseminated disease in the newborn is prevented by IFN-β treatment. (A) Newborn WT mice were inoculated i.p. with 10^5^ PFU of HSV-1 (KOS) or mock infected. Serum and brain were collected 1 h after 2 mM NaF i.p. injection at the indicated time points, and the blood-brain barrier permeability index was determined as the normalized ratio of brain fluorescence and serum fluorescence. (B) Newborn WT mice were inoculated i.p. with 10^5^ PFU of HSV-1 (KOS) and given daily doses of 10^4^ IU of recombinant murine IFN-β or vehicle control (PBS) starting the day before infection. The blood-brain barrier permeability index was determined using the same procedure. (C) Model for IFN-dependent increased susceptibility to HSV-1 infection in the newborn. (*, *P* ≤ 0.05; **, *P* ≤ 0.01; ***, *P* ≤ 0.001; ****, *P* ≤ 0.0001. All error bars represent SEM.)

The delay in HSV-1 spreading to the newborn CNS during disseminated disease led us to investigate how IFN-β treatment affects the blood-brain barrier (BBB). Although IFN-β is used in MS for its effects as an immunomodulator, mounting evidence suggests that the stabilization of the BBB is also crucial for IFN-β effectiveness in controlling MS symptoms (33, 34). IFN-β also was shown to play an important role in BBB modulation during infection of West Nile virus infection of the CNS after hematogenous dissemination (35–37). We first studied whether disseminated HSV-1 infection led to increased permeability of the BBB in the newborn. To study BBB permeability, we used an assay based on the infiltration of sodium fluorescein (NaF) from the periphery to the CNS and determined a BBB permeability index by normalizing fluorescein intensity in the brain parenchyma to fluorescein intensity in the serum. We found that disseminated HSV-1 infection significantly increases BBB permeability 1 day postinfection and continues to increase as infection progresses (Fig. 6A).

We next determined whether IFN-β treatment alters BBB permeability in the context of disseminated HSV-1 infection. There was no significant difference in BBB permeability during the early stages of infection, although we saw a trend at day three postinfection. By 5 days postinfection, IFN-β treatment significantly stabilized the BBB (Fig. 6B). These results suggest that peripheral IFN-β treatment prevents the breakdown of the blood-brain barrier during disseminated HSV-1 infection in the newborn and may explain, in part, the neuroprotective effects of IFN-β treatment in newborns.

## DISCUSSION

Neonatal HSV-1 infections commonly result in life-threatening encephalitis or disseminated disease (5). This is in stark contrast to adult HSV-1 infections, which are largely asymptomatic or result in benign mucocutaneous disease. Several studies have aimed at studying age-dependent differences in the immune response to HSV-1 between newborns and adults (17, 38–43), but our understanding of the significant difference in disease outcomes in this population remains limited. Several promising candidates for an effective HSV vaccine have failed to show efficacy in clinical trials (44, 45). For this reason, alternative strategies focusing on modulating the immune response to neonatal HSV-1 infection have been proposed (42, 46, 47). The development of effective immunomodulatory therapies for neonatal HSV-1 infection requires a deeper understanding of the mechanisms involved in mediating the distinct immune responses found in the newborn.

In our study, we used a murine model to study age-dependent differences in the type I IFN response to HSV-1 infection. Our model replicated the age-dependent susceptibility to HSV-1 infection observed in humans, as we previously demonstrated, showing a clear survival advantage in the adult relative to newborns (39). Importantly, these previous studies done with 129s mice suggested no survival advantage in WT newborns compared to newborns lacking IFNAR1. However, our results suggest that in C57BL/6j mice IFNAR1 signaling prolongs survival but still fails to protect the newborn from HSV-1 CNS infection. This highlights important, previously described differences in the immune response between commonly used laboratory mouse strains (48).

TLR levels are tightly regulated in the brain during development, as they have been shown to regulate neurogenesis (18, 49), which can have important implications in the immune response to viral infection of the CNS during the early stages of life, making newborns particularly susceptible to pathogens that infect the CNS. Our results suggest that the levels of several other components of the type I IFN response are differentially modulated in the newborn brain throughout development, suggesting an immunologically distinct environment. These differences do not necessarily reflect a global downregulation of this pathway, as some components, such as TLR-3 and STAT1, were found to be higher in the newborn brain, while IFNAR1 and PKR were downregulated. Notably, our findings showing TLR-3 being upregulated in the newborn brain correspond with previous studies indicating the critical role for TLR-3 in neurodevelopment (18, 50, 51). Our data also demonstrate that the transition from the newborn to the adult innate immune signaling profile does not switch over immediately after the newborn period but rather follows a progressive development until around weaning age.

Intrinsic and cell-mediated innate immune responses (52, 53), as well as the adaptive immune response (54, 55), have been shown to be involved in controlling initial HSV replication during acute infection, regulating the establishment of latency and, later, virus reactivation. The apparent immunodeficiency observed in the newborn has largely been attributed to the lack of antigenic exposure resulting in ineffective B and T cell-mediated immunity, as well as their reliance on passive immunity (56, 57). However, more data are accumulating to suggest several distinct differences in the newborn immune response to HSV infection that lead to increased severity and susceptibility to disease compared to that of adults (42). Although many of these studies have investigated the mechanisms involved in modulating the distinct adaptive immune responses observed in the newborn, little is known about the regulation of the innate immune response during the early stages of development. Studies looking at the distinct bias for Th2 over Th1 responses in the newborn have shown that these loci are epigenetically regulated during development and favor the expression of Th2-associated genes (58–61). These findings provide an interesting hypothesis for the regulation of the type I IFN response at the transcription level that could account for the protein-level differences we found in our studies. Importantly, all of these findings seem to reflect a careful balance in the regulation of the immune response during the early stages of development. These differences may be crucial for proper neurodevelopment (62–65) and, as more recent studies have suggested, the establishment of the microbiome by regulating the colonization of multiple commensal organisms (66–68).

Early work focusing on the increased susceptibility of newborn mice to HSV infection suggested that the transfer of interferon-stimulated cells had protective effects on the newborn (69, 70). However, there was insufficient understanding of the effects these cells or the increased interferon levels have beyond a survival advantage in infected newborn mice. Here, we show that supplementing the low IFN-β production in newborns by treatment with recombinant murine IFN-β resulted in both reduced replication in the periphery and direct effects on immune signaling in the CNS of treated mice (Fig. 5). IFN-β treatment induced the upregulation of cGAS, an important interferon-stimulated gene in the immune response to HSV-1 (8), in the newborn brain parenchyma. While changes in IFNAR1 and STAT1 were not statistically significant, the trending increase could be a reflection of the complex dynamics governing the regulation of these innate immune sensors when IFNAR signaling is initiated (20, 21). These results suggest that peripheral IFN-β treatment has a direct effect modulating the type I IFN response in the brain and induces an antiviral state. These data suggest that while decreased replication in the periphery could contribute to increased survival, the observed changes in the type I IFN response in the brain parenchyma also likely contribute to controlling viral replication in the CNS and increased survival.

HSV entry into the CNS during neonatal infection has been shown to be most commonly the result of hematogenous spread, which suggests a direct involvement of the BBB in neonatal HSV pathogenesis (22). The BBB is composed mainly of endothelial cells that express multiple tight-junction proteins as well as other supporting cells, such as pericytes and the end-feet of astrocytes (71). It plays an important role during viral infection both in preventing neuroinvasion and regulating the infiltration of immune cells into the brain (72, 73). Previous studies have shown that the BBB is compromised during HSE following active viral replication in the brain parenchyma, but little was known about the dynamics of this process (74–77). Here, we demonstrate that BBB permeability increases as early as 1 day postinfection in the newborn during disseminated disease, as evidenced by our sodium fluorescein assay. Furthermore, our results suggest that beyond its role in regulating the type IFN response in the brain parenchyma, IFN-β treatment has a direct effect in stabilizing the BBB during disseminated infection in the newborn, resulting in delayed neuroinvasion. While the baseline permeability of the BBB is similar between newborns and adults, our data suggest that the increased susceptibility of the newborn brain to viral neuroinvasion is due to the dynamic immune regulation of the BBB during infection.

Previous studies have shown that the choroid plexus, a specialized epithelium in the brain ventricles responsible for producing the cerebrospinal fluid (CSF), has an age-dependent susceptibility to HSV-1 infection (17). Adult choroid plexus is resistant to HSV-1 infection, while the newborn is highly susceptible. Importantly, this age-dependent tissue-specific susceptibility to infection was found to be dependent on IFNAR1 expression. These findings suggest that the blood-CSF barrier also is involved in regulating the entry of HSV-1 into the brain. In this case, direct infection of choroid plexus epithelial cells also could be a mechanism for entry into the brain. The reliance on IFNAR signaling for resistance to HSV-1 suggests that IFN-β treatment in our study is also protecting other specific tissues, such as the choroid plexus, from becoming infected in the newborn. While our study demonstrates dynamic changes to the BBB during IFN treatment, it is likely that IFN is also influencing viral replication in other tissues.

Overall, our studies reveal important differences in the type I IFN response in the brains of newborn and adult mice. However, more rigorous and high-throughput approaches are necessary to understand the complexity of the differences in the immune response between these two age groups. Importantly, although our model seems to reflect the age-dependent susceptibility to HSV observed in humans, mouse models have significant limitations when it comes to translating findings for human applications (78).

Finally, our results suggest a model (Fig. 6C) in which increased susceptibility to HSV-1 infection in newborns is associated with insufficient type I IFN signaling. Low levels of IFN-β production in response to infection combined with lower basal levels of IFNAR1 in the newborn lead to impaired type I IFN signaling, resulting in the breakdown of the BBB, rapid spread of HSV-1 to the CNS, and uncontrolled viral replication in the brain parenchyma. In the case of the adult, where IFN-β production is rapidly induced, or when IFN-β is supplemented to the newborn, HSV-1 neuroinvasion is prevented by the stabilization of the BBB, and increased type I IFN signaling in the CNS results in the clearance of the virus from the brain. The protective adult phenotype is recapitulated in the newborn with IFN-β supplementation in our model. Work from others has shown that the T-cell response in newborns can be stimulated to produce a more Th1-weighted response, as seen in the adult (79, 80). Our data suggest that innate immune signaling pathways can be similarly modulated to drive an adult immune phenotype in the newborn brain. As a result, our studies denote a potential new strategy for the treatment of neonatal HSV-1 infection by modulation of the innate immune response and also serve as a basis to further our understanding of the intrinsic differences in the innate immune response between the newborn and the adult.

## MATERIALS AND METHODS

### Viruses and cells.

The HSV-1 strain KOS was obtained from laboratory stocks compiled by Patricia Spear (Northwestern University, Chicago, IL). Vero cells (CCL-81; ATCC) were used to propagate and titer the virus. Cells were tested for mycoplasma using the MycoAlert mycoplasma detection kit (LT07-118; Lonza). Vero cells were cultured in Dulbecco’s modified Eagle medium (DMEM) plus 10% (vol/vol) fetal bovine serum and 1% penicillin-streptomycin. Plaque titrations were performed using standard serial dilution methods. Viral stocks were diluted to the appropriate concentration in PBS containing 1% glucose and 1% heat-inactivated serum.

### Murine model of HSV infection.

C57BL/6J (WT; 000664) and B6(Cg)-*Ifnar1^tm1.2Ees^*/J (IFNARKO; 028288) mice were purchased from Jackson Laboratories and subsequently bred at Northwestern University. Adult male mice were inoculated at 8 to 10 weeks of age, and newborn mice were inoculated at 7 days of age, which immunologically corresponds most closely to humans at birth (58). Only adult males were used for survival experiments to preserve female breeders needed to obtain newborn mice used in experiments. Sex was not determined for newborn mice due to their young age.

For intracranial (i.c.) inoculations, either adult or newborn mice were inoculated with 10^4^ PFU in a total volume of 10 μl using a 25-μl positive displacement syringe (80401; Hamilton Company) and a 26-gauge needle. The same dose of virus was used based on the similar brain size between newborns and adults. The needle was placed in the approximate region of the hippocampus, equidistant between the lambda and bregma, through the left parietal bone lateral to the sagittal suture. Infected mice were monitored daily for signs of disease and their weight recorded. Those displaying severe symptoms, or 30% weight loss, were immediately euthanized. Brain tissue was collected at the indicated time points or experimental endpoint (14 days postinfection).

For intraperitoneal (i.p.) inoculations, newborn mice were inoculated with 10^5^ PFU of HSV-1 in a total volume of 100 μl using a 1-ml U-100 syringe (28 gauge by 1/2 inch). Infected mice were monitored daily for signs of disease and their weight recorded. Those displaying severe symptoms, or 30% weight loss, were immediately euthanized. Lung, liver, spleen, and brain tissues were collected at the indicated time points or experimental endpoint (14 days postinfection). Tissues were weighed and homogenized in DMEM with 1% penicillin-streptomycin and sonicated.

### Immunoblots.

Brain tissue was collected from newborn and adult mice and homogenized in T-PER tissue protein extraction reagent (78510; ThermoFisher) supplemented with Halt protease and phosphatase inhibitor cocktail (78440; ThermoFisher). Western blot analyses were performed on tissue lysates using antibodies against IFNAR1 (ab124764; Abcam), PKR (ab45427; Abcam), cGAS (ABF124; Millipore), TLR-3 (6961; Cell Signaling), STAT1 (9172; Cell Signaling), and an anti-glyceraldehyde-3-phosphate dehydrogenase (GAPDH) antibody (ab8245; Abcam) as a loading control. A 1:1,000 dilution was used for all primary antibodies. Blots were visualized using the LI-COR Odyssey system, and densitometric analysis was performed using LI-COR Image Studio Lite 5.2.5.

### Quantitative RT-PCR.

Total brain RNA was extracted using TRIzol reagent (15596026; ThermoFisher) according to the manufacturer’s specifications. cDNA was made using a high-capacity cDNA reverse transcription kit (4368814; Applied Biosystems). Quantitative PCR was performed using PowerUp SYBR green master mix (A25742; ThermoFisher) and the Step One Plus real-time PCR system (Applied Biosystems) by following the manufacturer’s specifications. Transcript levels were normalized to those of *gadph*. The following primers were used: *ifnb* sense, 5′-AAGAGTTACACTGCCTTTGCCATC-3′; *ifnb* antisense, 5′-CACTGTCTGCTGGTGGAGTTCATC-3′; *gapdh* sense, 5′-TGGTATCGTGGAAGGACTCATGAC-3′; and *gapdh* antisense, 5′-ATGCCAGTGAGCTTCCCGTTCAGC-3′.

### Recombinant IFN-β treatment.

Recombinant mouse IFN-beta protein (8234-MB-010; R&D Systems) was reconstituted in endotoxin-free Dulbecco’s PBS (TMS-012-A; Millipore) plus 0.1% bovine serum albumin. Newborn mice were treated daily with 10,000 IU IFN-β starting at 6 days of age and i.p. infected with HSV-1 at 7 days of age. IFN-β was administered intraperitoneally in a volume of 100 μl using a 1-ml U-100 syringe (28 gauge by 1/2 inch).

### Blood-brain barrier permeability assay.

The protocol used to assess blood-brain barrier permeability was adapted from the protocol previously described by Devraj et al. (81). Briefly, 100 μl 2 mM sodium fluorescein (F6377-100G; Sigma) was administered intraperitoneally to newborn mice at the indicated time points using a 1-ml U-100 syringe (28 gauge by 1/2 inch). After 1 h, blood was collected using heparinized capillaries, and brain tissue was collected following perfusion with cold PBS. Blood samples were centrifuged, and serum was collected for fluorescein intensity measurement. Brain tissue was flash frozen and then thawed and homogenized in sterile PBS. Homogenized brain samples were centrifuged, and the supernatant was collected for fluorescein intensity measurement. Fluorescein intensity was measured in a VICTOR Nivo multimode microplate reader (Perkin Elmer) at an excitation wavelength of 480 nm and an emission wavelength of 530 nm. Serum and brain samples from sham-treated newborn mice were used as blanks for all samples. The blood-brain barrier permeability index was calculated, using raw fluorescence units (RFUs), as permeability index (ml/g) = (tissue RFUs/g tissue weight)/(serum RFUs/ml serum).

### Statistics.

All statistical analyses were performed using GraphPad Prism 7. Survival curves were analyzed using log rank (Mantel-Cox) test. Densitometry data were analyzed using a one-way analysis of variance and the Holman-Sidak multiple-comparison test. All other data were analyzed using two-tailed unpaired Student's *t* test. Log-transformed values were used to analyze viral titers. *, *P* ≤ 0.05; **, *P* ≤ 0.01; ***, *P* ≤ 0.001; ****, *P* ≤ 0.0001. All error bars represent standard errors of the means (SEM).

### Study approval.

Animal care and use in this study were in accordance with institutional and NIH guidelines, and all studies were approved by the Northwestern University Animal Care and Use Committee.
